# Length of Hospital Stay for Hip Fracture and 30-Day Mortality in People With Alzheimer’s Disease: A Cohort Study in Finland

**DOI:** 10.1093/gerona/glaa199

**Published:** 2020-08-14

**Authors:** Piia Lavikainen, Marjaana Koponen, Heidi Taipale, Antti Tanskanen, Jari Tiihonen, Sirpa Hartikainen, Anna-Maija Tolppanen

**Affiliations:** 1 School of Pharmacy, University of Eastern Finland, Kuopio, Finland; 2 Kuopio Research Centre of Geriatric Care, University of Eastern Finland, Finland; 3 Centre for Medicine Use and Safety, Faculty of Pharmacy and Pharmaceutical Sciences, Monash University, Melbourne, Victoria, Australia; 4 Department of Clinical Neuroscience, Center for Psychiatry Research, Karolinska Institutet and Stockholm County Council, Sweden; 5 Department of Forensic Psychiatry, Niuvanniemi Hospital, University of Eastern Finland, Kuopio, Finland

**Keywords:** Community dwellers, Death, Dementia, In-hospital days

## Abstract

**Background:**

Persons with Alzheimer’s disease (AD) are at higher risk of hip fractures (HFs) than general older population and have worse prognosis after HF. Hospital stays after HF have shortened along time. We investigated the association between length of hospital stay after HF and mortality after discharge among persons with AD.

**Method:**

The MEDALZ cohort includes all Finnish community dwellers who received clinically verified AD diagnosis in 2005–2011 (*N* = 70 718). Patients who experienced first HF after AD diagnosis in 2005‒2015 (*n* = 6999) were selected. Length of hospital stay for HF was measured as a sum of the consecutive days spent in hospital after HF until discharge. Outcome was defined as death within 30 days after hospital discharge.

**Results:**

Mean of overall length of hospital stay after a HF decreased from 52.6 (*SD* 62.9) days in 2005 to 19.6 (*SD* 23.1) days in 2015. Shortest treatment decile (1‒4 days) had the highest risk of death within 30 days after discharge (adjusted hazard ratio [aHR] 2.76; 95% confidence interval [CI] 1.66–4.60) in addition to second (5‒6 days; aHR 2.52; 95% CI 1.50–4.23) and third (7‒10 days; aHR 2.22; 95% CI 1.34–3.69) deciles when compared to the sixth decile of length of stays (21‒26 days).

**Conclusions:**

Among persons with AD, shorter length of hospital stay after HF was associated with an increased risk of death after discharge. After acute HF treatment, inpatient rehabilitation or proper care and services in home need to be organized to older persons with AD.

Hip fractures are the most common fractures among older population and constitute high morbidity and mortality (1). In Finland, 5.7% of hip fracture patients had died within 1 month, 11.9% within 3 months, and 18.4% within 1 year of the fracture in 2013 (2). In addition, hip fractures increase the further use of health care services for 1–2 years after the hip fracture, and result in significant clinical and economic burden and disability (3–5). Persons with Alzheimer’s disease (AD) are at higher risk of hip fractures than persons without cognitive disorder (4,6). They also have worse prognosis after hip fracture because of greater challenges in rehabilitation (7) and higher post-fracture mortality (4,8).

Hip fracture treatment targets to preserve patient’s level of functioning at the same level as before hip fracture (9). In Finland, all hip fractures are treated at hospital inpatient wards and include surgical treatment. Typical hospital stay with hip fracture is few days and thereafter patient is transferred for rehabilitation to a community hospital (10). Typically, patient is discharged to home after 2–4 weeks of hip fracture occurrence (3). Hospital stays both in general and due to hip fractures are longer among patients with dementia than without cognitive disorder (11).

Hospital stays after hip fracture surgery have shortened along 20th century (3,12–16). Conflicting findings of the association between length of hospital stay after hip fracture and risk of short-term death after hospital discharge in the general population aged 50 years and older are reported from Europe and the United States (15,16). However, there are no prior studies investigating these associations among vulnerable population with cognitive disorders and especially in persons with AD, who have a higher predisposition to both hip fracture and adverse outcomes after it. Therefore, we investigated the association between length of hospital stay after hip fracture and short-term mortality after hospital discharge among persons with AD.

## Method

We used data from the Finnish MEDALZ (MEDication use and ALZheimer’s disease) cohort (17). The cohort includes 70 718 community-dwelling persons who received clinically confirmed AD diagnosis in 2005‒2011 in Finland. The data have been linked across several administrative health registers. The Finnish Care Register for Health Care (1972‒2015) maintained by the National Institute for Health and Welfare includes data on hospital admissions and their diagnostic and procedure data. The International Classification of Diseases, 10th revision (ICD-10) (18) was in use during the study period. Death dates (2005‒2015) were obtained from the Cause of Death Register maintained by the Statistics Finland. The Finnish Prescription Register (1995‒2015) maintained by the Social Insurance Institution (SII) contains information on reimbursed medication purchases for all Finnish residents, such as dispensing date, Anatomical Therapeutic Chemical (ATC) classification code (19) of the purchase, strength, and the quantity dispensed. However, medications used in the hospitals are not recorded in the Prescription Register. The Special Reimbursement Register (1972‒2015) maintained by the SII contains information on entitlements to higher medication reimbursement due to chronic diseases, such as AD.

We utilized data from the Finnish Prescription Register to model medication use periods with our previously established and validated Prescriptions to Drug Use Periods (PRE2DUP) method (20,21). Briefly, the method estimates medication use periods based on drug dispensing data and accounts for stockpiling of medications, individual medication purchase patterns as well as interruptions in medication purchases due to in-hospital and institutional stays.

Register data were linked applying personal identification numbers. We used only de-identified data and thus, according to Finnish law, ethical approval was not needed.

### Study Population

Persons experiencing hip fractures were identified from the Finnish Care Register for Health Care with ICD-10 codes (years 1996‒2015) S72.0, S72.1, and S72.2. For identification of previous hip fractures, ICD-9 codes (1987‒1995) 820 and ICD-8 codes (1972‒1986) 82000, 82010, 82090, 82001, 82011, and 82091 as primary or secondary diagnosis were utilized.

### Exposure

Length of hospital stay was defined as a sum of the consecutive days (with allowance of 1-day interruption, ie, if discharge occurred at May 22nd and the next admission at May 23rd, they were considered to belong to the same treatment episode) spent in hospital after hip fracture until discharge measured from the Finnish Care Register for Health Care. Calculation of the sum of consecutive hospital days was started at the date of admission because the actual day of hip fracture occurrence is not recorded in the register data. This approach has been previously used to monitor the performance of hip fracture treatment in Finland (13,22,23).

In secondary analyses, we calculated hospital days including only stays in central and university hospitals and thus, excluding stays in community hospitals. Persons discharged to community hospitals are in a poorer health status or have complications compared to those discharged to home. In the Finnish Care Register for Health Care, service producers are registered, recorded and, further, classified by type of service. Here, we excluded hospital stays where the type of service indicated that the patient stayed in community hospital.

### Outcome

All-cause deaths within 30 days after hospital discharge were identified from the Cause of Death Register. In sensitivity analysis, the follow-up for deaths was extended to 90 days.

### Covariates

The covariates were chosen based on previously documented associations with fracture (3), length of hospital stay after hip fracture (24), or death. The analyses were adjusted for use of benzodiazepines and related drugs, antidepressants, and antipsychotics, history of coronary artery disease or stroke, diabetes, asthma or chronic obstructive pulmonary disease, renal failure, and any cancer measured on the date of hospital discharge. In addition, the models were adjusted for sociodemographic factors, such as age, sex, time since AD diagnosis, university hospital catchment area, and occupational socioeconomic class.

The models were also adjusted for covariates describing type of hip fracture as well as functionality and frailty of the patient. Type of hip fracture was classified as a fracture of neck of femur (ICD-10 code S72.0), pertrochanteric fracture (S72.1), and subtrochanteric fracture (S72.2). From the hospital discharge data, we were able to identify whether a patient was admitted from community-based living facilities, such as nursing homes, or home. In addition, required level of assistance at discharge was also recorded and classified as “independent or nearly independent,” “intermittent need,” “recurrent need,” “nearly continuous need,” and “continuous need.” This was considered to be a proxy of patient’s functional status. Data on required level of assistance was missing from 266 (4.2%) persons alive at discharge, but missing values were classified to “data missing“, and included in statistical analyses. Definitions of and classifications for covariates are presented more detail in Supplementary Table 1.

### Statistical Analyses

**Figure 1.**
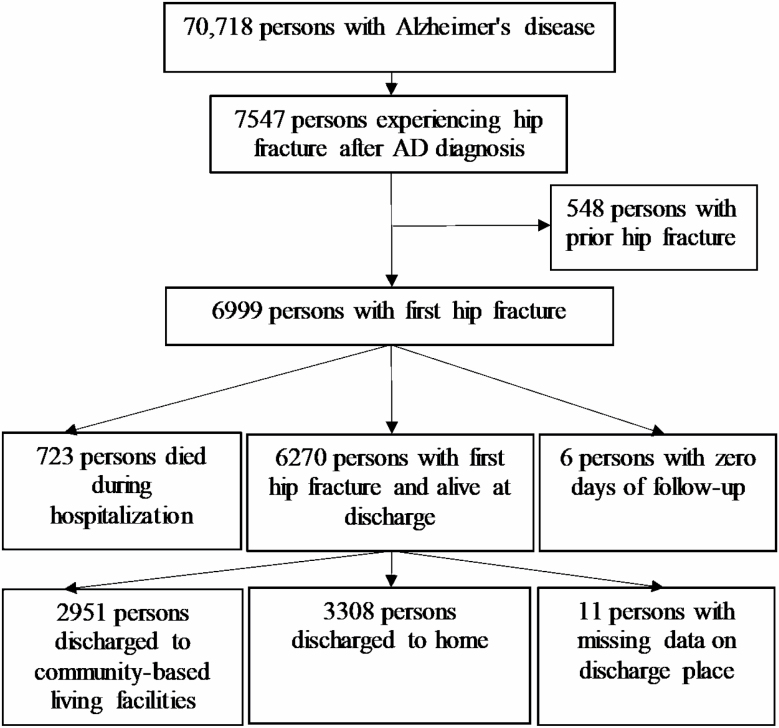
Flow chart of persons with first hip fracture after diagnosis of Alzheimer’s disease.

We identified 7547 persons who experienced hip fracture after AD diagnosis in 2005‒2015 (Figure 1). Of those, 548 had a history of hip fracture prior to AD diagnosis and 6 had zero days of follow-up due to admission at the date of administrative end of follow-up in December 31, 2015 and thus, they were excluded from the further analyses. Differences in baseline characteristics between persons discharged to community-based living facilities or home were examined using standardized difference that is independent of sample size (25). Standardized difference values >0.1 were considered to indicate meaningful systematic differences between the groups.

Due to non-linear U-shaped association observed in graphical examination of hazard ratio (HR) of short-term mortality by length of hospital stay after hip fracture, length of hospital stay was divided into fractiles (here, deciles or quintiles). Fractile with the lowest risk was selected as a reference group (26). In primary analyses, the length of hospital stay was divided into deciles. In secondary analyses, where length of hospital stay was recalculated excluding stays in community hospitals, length of hospital stay was divided into quintiles due to shorter overall hospital stays than in the primary analyses. Associations between fractiles of length of hospital stay and death were assessed using Cox’s proportional hazards regression models. Follow-up ended in death, end of 30-day follow-up period or administrative end of study at December 31, 2015. Persons who died during the hospital stay were excluded from these analyses. In the primary analysis, risk of mortality was lowest in the 10th decile, but due to large variation in hospital days in the 10th decile, the decile with the second lowest mortality risk (6th decile) was selected as a reference group. In the secondary analysis, third quintile had the lowest mortality risk and was selected as a reference group. Analyses were executed as unadjusted and adjusted for the covariates in the Table 1 as well as admission year. Assumption of proportional hazards was assessed with exploring parallelism of log negative and log estimated survival curves for each covariate. To assess whether the association was modified by use of antidepressants and antipsychotics, a model with interaction term (length of stay*antidepressant or antipsychotic use) was fitted, but as there was no evidence for interaction (*p* = .6988) subgroup analyses were not performed.

**Table 1.** Baseline (at the time of discharge from hospital care) Characteristics of Alzheimer’s Disease Patients With First Hip Fracture

	Persons With First Hip Fracture (*n* = 6993)	Persons Died During Hospital Stay (*n* = 723)	Persons Alive at Discharge (*n* = 6270)	Discharged to Community-Based Living Facilities (*n* = 2951)	Discharged to Home (*n* = 3308)	*SD* ^a^
	*n* (%)	*n* (%)	*n* (%)	*n* (%)	*n* (%)	
Age (y), mean (*SD*)	84.5 (6.2)	86.3 (5.6)	84.3 (6.2)	84.6 (6.1)	84.1 (6.3)	0.089
Age, classified						
<75 y	480 (6.9)	22 (3.0)	458 (7.3)	193 (6.5)	265 (8.0)	0.057
75‒84 y	2974 (42.5)	250 (34.6)	2724 (43.4)	1267 (42.9)	1452 (43.9)	0.019
≥85 y	3539 (50.6)	451 (62.4)	3088 (49.3)	1491 (50.5)	1591 (48.1)	0.049
Female	5244 (75.0)	424 (58.6)	4820 (76.9)	2202 (74.6)	2608 (78.8)	0.100
Occupational socioeconomic position						
Managerial/professional	2957 (42.3)	314 (43.4)	2643 (42.2)	1232 (41.8)	1407 (42.5)	0.016
Office	1248 (17.9)	147 (20.3)	1101 (17.6)	513 (17.4)	586 (17.7)	0.009
Farming/forestry	671 (9.6)	48 (6.6)	623 (9.9)	307 (10.4)	314 (9.5)	0.030
Sales, industrial, cleaning	1302 (18.6)	127 (17.6)	1175 (18.7)	540 (18.3)	634 (19.2)	0.022
Unknown, no response	815 (11.7)	87 (12.0)	728 (11.6)	359 (12.2)	367 (11.1)	0.033
Time since diagnosis of Alzheimer’s disease (d), mean (*SD*)	1176.3 (781.8)	1223.8 (769.3)	1170.8 (784.8)	1120.7 (756.2)	1215.6 (805.8)	0.121
Benzodiazepines and related drugs	2337 (33.4)	239 (33.1)	2098 (33.5)	923 (31.3)	1171 (35.4)	0.088
Antipsychotics	2377 (34.0)	255 (35.3)	2122 (33.8)	941 (31.9)	1175 (35.5)	0.077
Antidepressants	2471 (35.3)	218 (30.2)	2253 (35.9)	996 (33.8)	1253 (37.9)	0.086
Coronary artery disease	2201 (31.5)	300 (41.5)	1901 (30.3)	930 (31.5)	971 (29.4)	0.047
Stroke	950 (13.6)	127 (17.6)	823 (13.1)	412 (14.0)	411 (12.4)	0.045
Diabetes	933 (13.3)	99 (13.7)	834 (13.3)	422 (14.3)	410 (12.4)	0.045
Asthma/COPD	792 (11.3)	116 (16.0)	676 (10.8)	327 (11.1)	349 (10.6)	0.017
Renal failure	114 (1.6)	17 (2.4)	97 (1.6)	41 (1.4)	56 (1.7)	0.045
Any cancer	1003 (14.3)	126 (17.4)	877 (14.0)	422 (14.3)	454 (13.7)	0.017
Required level of assistance						
Independent or nearly independent	296 (4.3)	0	296 (4.7)	48 (1.6)	247 (7.5)	0.283
Intermittent need	1068 (15.3)	0	1068 (17.0)	267 (9.1)	801 (24.2)	0.416
Recurrent need	2222 (31.8)	1 (0.1)	2221 (35.4)	1112 (37.7)	1104 (33.4)	0.090
Nearly continuous need	1105 (15.8)	4 (0.6)	1101 (17.6)	647 (21.9)	452 (13.7)	0.217
Continuous need	1324 (18.9)	6 (0.8)	1318 (21.0)	736 (24.9)	580 (17.5)	0.182
Data missing	978 (14.0)	712 (98.5)	266 (4.2)	141 (4.8)	124 (3.7)	0.051
Type of hip fracture						
Fracture of neck of femur	4352 (62.2)	445 (61.6)	3907 (62.3)	1810 (61.3)	2087 (63.1)	0.036
Pertrochanteric fracture	2222 (31.8)	239 (33.0)	1983 (31.6)	952 (32.3)	1029 (31.1)	0.025
Subtrochanteric fracture	420 (6.0)	39 (5.4)	381 (6.1)	189 (6.4)	192 (5.8)	0.025
Place of stay at admission						
* *Community-based living facilities	1671 (23.9)	202 (27.9)	1469 (23.4)	829 (28.1)	639 (19.3)	0.207
* *Home	5322 (76.1)	521 (72.1)	4801 (76.6)	2122 (71.9)	2669 (80.7)	0.207

*Notes*: COPD = chronic obstructive pulmonary disease.

^a^For the difference between patients discharged to community-based living facilities and home.

We performed several sensitivity analyses. First, we recalculated length of hospital stay including only hospital stays that included a hip fracture surgery identified with NOMESCO classification of surgical procedures codes (27) NFB10, NFB20, NFB30, NFB40, NFB50, NFJ50, NFJ52, and NFJ54. Second, we categorized length of hospital stay according to one used in previous publications (16,17) to aid in comparison of results (1–5, 6–10, 11–14, and ≥15 days). We performed 4-class analyses stratified by sex and by place of stay after hospital discharge (community-based living facilities or home). Third, to investigate whether the association remained similar during 2005–2015, we categorized the length of hospital stay to 1–10 and >10 days of stays and examined association with death stratified by admission year. Fourth, we replicated the primary analysis extending the follow-up of deaths to 90 days as a sensitivity analysis against the follow-up time. Fifth, we restricted the cohort to those who were alive on day 27 after admission and had length of hospital stay <27 days and examined the association between the first 6 deciles and death during 27–60 days after admission to account for an increased risk of death initially after the hip fracture.

All the analyses were conducted with SAS version 9.4 (SAS Institute Inc., Cary, North Carolina).

## Results

We identified 6993 AD persons with first hip fracture whose mean age was 84.5 years (*SD* 6.2) and 75.0% were female (Table 1). Altogether 723 persons died during the hospitalization after hip fracture. Of those who survived to discharge (*n* = 6270), 2951 persons (47.1%) were discharged to community-based living facilities, such as nursing homes, and 3308 persons (52.8%) to home (11 persons had missing data). Persons discharged to home were more often independent or required intermittent assistance compared with persons discharged to community-based living facilities (Table 1). In addition, persons discharged to home were more often women and had longer time since AD diagnosis at the time of discharge than those discharged to community-based living facilities. Characteristics by deciles of length of stay are shown in Supplementary Table 2. Those discharged after 1–4 for days of hospital stay were more likely to be discharged to community-based living facilities than those discharged after 11–15 days of hospital stay. 

Mean length of hospital stay after a hip fracture among persons alive at discharge decreased from 52.9 (*SD* 63.3) days in 2005 to 19.9 (*SD* 23.5) days in 2015 with an average of 36.1 (*SD* 58.9) days over time (Table 2). Persons discharged to home stayed longer in hospital after a hip fracture than persons discharged to community-based living facilities (39.9 vs 31.8 days, correspondingly, *p* < .001).

**Table 2.** Number of Hospital Admissions, Average Lengths of Hospital Stays, and Proportion of Deaths by Admission Year Among Alzheimer’s Disease Patients With First Hip Fracture

Admission Year	2005	2006	2007	2008	2009	2010	2011	2012	2013	2014	2015	Total
Among persons who died during hospital stay (*n* = 723)												
No. of admissions	1	23	42	64	99	88	99	90	94	72	51	723
LOS (d), mean (*SD*)	31	28.6 (56.3)	81.0 (304.4)	53.4 (71.3)	60.1 (172.2)	55.8 (130.2)	50.0 (117.3)	62.8 (157.5)	40.8 (104.6)	27.9 (35.0)	15.6 (18.3)	49.3 (136.4)
LOS (d), median (IQR)	31 (31‒31)	8 (3‒33)	9.5 (4‒41)	20.5 (6‒64.5)	18 (6‒58)	19.5 (7‒40.5)	19 (5‒47)	14 (5‒34)	15 (6‒43)	14.5 (6‒39)	11 (3‒26)	15 (5‒43)
Among persons alive at discharge (*n* = 6270)												
No. of admissions	69	241	360	543	590	759	894	902	797	610	505	6270
LOS (d), mean (*SD*)	52.9 (63.3)	48.0 (75.7)	49.6 (99.7)	38.9 (46.4)	42.0 (74.0)	37.4 (61.7)	36.9 (65.6)	36.0 (48.8)	32.7 (44.6)	28.1 (42.6)	19.9 (23.5)	36.1 (58.9)
LOS (d), median (IQR)	33 (13‒59)	31 (13‒52)	26 (9‒54.5)	24 (9‒47)	25 (10‒48)	23 (10‒44)	20.5 (8‒41)	21 (8‒41)	19 (8‒36)	16 (7‒32)	12 (5‒25)	21 (8‒41)
No. (%) of deaths within 30 d after discharge	0 (0.0)	7 (2.9)	12 (3.3)	25 (4.6)	38 (6.4)	35 (4.6)	50 (5.6)	49 (5.4)	54 (6.8)	47 (7.7)	36 (7.1)	353 (5.6)
Among persons alive at discharge who were discharged to community-based living facilities (*n* = 2951)												
No. of admissions	31	139	206	306	312	372	427	397	324	224	213	2951
LOS (d), mean (*SD*)	47.1 (72.7)	48.8 (95.8)	51.2 (128.1)	33.9 (51.4)	38.2 (94.7)	31.5 (76.1)	31.6 (50.9)	28.1 (49.2)	27.5 (46.1)	19.6 (35.2)	15.0 (25.8)	31.8 (68.8)
LOS (d), median (IQR)	17 (1‒51)	18 (8‒49)	13 (6‒49)	13 (6‒38)	12 (7‒36.5)	13 (6‒29.5)	10 (6‒28)	10 (5‒28)	9 (5‒28)	8 (4‒19.5)	6 (4‒12)	10 (5‒29)
No. (%) of deaths within 30 d after discharge	0 (0.0)	7 (5.0)	10 (4.9)	23 (7.5)	28 (9.0)	31 (8.4)	35 (8.2)	36 (9.1)	40 (12.4)	25 (11.2)	15 (7.0)	250 (8.5)
Among persons alive at discharge who were discharged to home (*n* = 3308)												
No. of admissions	38	102	147	237	278	387	467	505	473	386	288	3308
LOS (d), mean (*SD*)	57.7 (55.0)	47.0 (32.9)	47.8 (37.1)	45.4 (38.0)	46.3 (39.2)	43.0 (42.9)	41.8 (76.3)	42.2 (47.7)	36.3 (43.2)	33.0 (45.6)	23.5 (21.2)	39.9 (48.3)
LOS (d), median (IQR)	47 (26‒62)	38 (27‒56)	35 (24‒62)	35 (23‒54)	35 (22‒53)	31 (19‒51)	27 (17‒44)	28 (17‒49)	25 (13‒42)	20.5 (12‒38)	19 (10‒29)	28 (17‒47)
No. (%) of deaths within 30 d after discharge	0 (0.0)	0 (0.0)	2 (1.4)	2 (0.8)	10 (3.6)	4 (1.0)	15 (3.2)	13 (2.6)	14 (3.0)	22 (5.7)	21 (7.3)	103 (3.1)

*Note*: IQR = interquartile range; LOS = length of stay.

**Figure 2.**
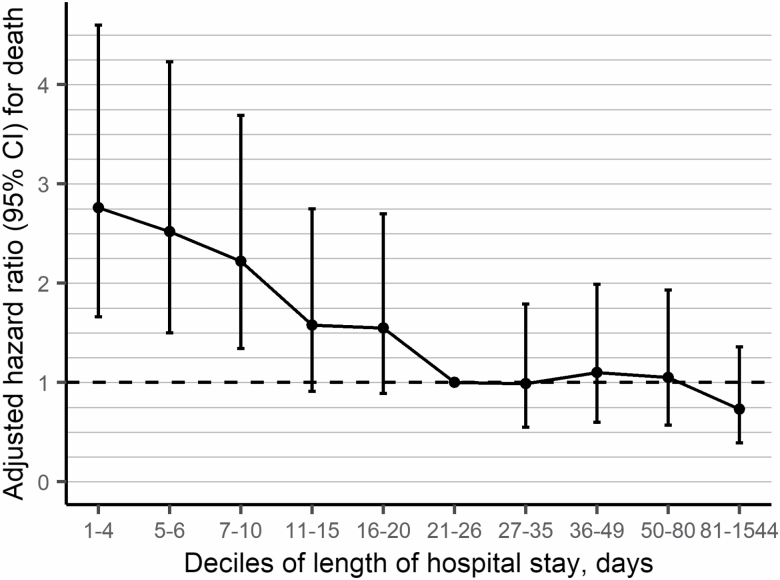
Adjusted hazard ratios for the association between deciles of length of hospital stay after hip fracture and mortality among persons with Alzheimer’s disease from the Cox regression model. Sixth decile (21–26 d) was a reference group. Vertical error bars show 95% confidence intervals. Analysis was adjusted for age, gender, occupational socioeconomic position, university hospital catchment area, time since diagnosis of Alzheimer’s disease, type of hip fracture, admission year, place of stay at admission, use of benzodiazepines and related drugs, antipsychotics, antidepressants, history of coronary artery disease, stroke, diabetes, asthma/chronic obstructive pulmonary disease, renal failure, any cancer, and required level of assistance after hospital discharge.

In total, 353 (5.6%) deaths occurred during the 30-day follow-up among those who survived to discharge (Table 2). Altogether, 8.5% of persons discharged to community-based living facilities died during the 30-day follow-up, whereas the corresponding proportion for those discharged to home was 3.1%. In primary analysis, shortest treatment decile (1‒4 days) had the highest risk of death within 30 days after discharge (adjusted HR [aHR] 2.76; 95% confidence interval [CI] 1.66–4.60) in addition to second (5‒6 days; aHR 2.52; 95% CI 1.50–4.23), and third (7‒10 days; aHR 2.22; 95% CI 1.34–3.69) deciles when compared to the sixth decile of length of stays (21‒26 days) (Figure 2; Supplementary Table 3).

In secondary analyses, length of hospital stay was recalculated excluding community hospital days (Supplementary Figure 1). Baseline characteristics are shown in Supplementary Table 4. Mean length of hospital stay excluding community hospital days among persons alive at discharge decreased from 8.8 (*SD* 5.6) days in 2005 to 5.2 (*SD* 2.9) days in 2015 (Supplementary Table 5). Hospital stays of 1–3 days as well as stays of 9–65 days were associated with an increased death risk (adjusted HR for 1–3 days 1.42, 95% CI 1.08‒1.88, adjusted HR for 9–65 days 1.55, 95% CI 1.18–2.04) when compared with stays of 5–6 days (Supplementary Figure 2 and Supplementary Table 3).

In sensitivity analysis restricted to only hospital stays including a hip fracture surgery, comparable results with our primary analysis were produced (Supplementary Table 6). When length of hospital stay including community hospital days was categorized to 4 classes, hospital stays of 11–14 days were associated with decreased death risk (HR 0.62, 95% CI 0.41–0.96) compared with stays of 1–5 days (Table 3). Hazard ratio was 0.38 (95% CI 0.29–0.50) for ≥15 days stays. No differences in associations were seen when the analysis was stratified by sex or by place of stay after hospital discharge (community-based living facilities or home) (Table 3). Associations of 1‒10 days hospital stays and 30-day mortality after discharge when compared with >10 days hospital stays stratified by admission years are presented in Supplementary Figure 3. We did not observe significant change in the strength of association between LOS and mortality in these sensitivity analyses. When hospital stay was recalculated excluding community hospital days in secondary analysis, 6–10 days hospital stays were associated with decreased death risk (HR 0.79, 95% CI 0.64–0.98), whereas stays of 11‒14 days were associated with increased death risk (HR 1.47, 95% CI 1.05–2.05) when compared with shorter stays of 1–5 days (Table 3). Replicating the primary analysis by extending the follow-up of deaths to 90 days instead of 30 days resulted in similar, although somewhat weaker, associations (Supplementary Table 7). Restricting the cohort to those who were alive on day 27 after admission and had length of hospital stay <27 days indicated that shorter hospital stays of 1–10 days are associated with an increased death risk during 27–60 days after admission when compared with 21–27 days stays (Supplementary Table 8).

**Table 3.** Sensitivity Analyses for Risk of Death in General and Stratified by Sex and Place of Stay Among Alzheimer’s Disease Patients With First Hip Fracture

	All Persons With First Hip Fracture	Men	Women	Place of Stay After Hospital Discharge	
				Community-Based Living Facilities	Home
	aHR* (95% CI)	aHR* (95% CI)	aHR* (95% CI)	aHR^a^ (95% CI)	aHR^a^ (95% CI)
Hospital stays including community hospital days					
	*n* = 6270	*n* = 1450	*n* = 4820	*n* = 2951	*n* = 3308
Length of stay					
* *1‒5 d	1.00 (reference)	1.00 (reference)	1.00 (reference)	1.00 (reference)	1.00 (reference)
* *6‒10 d	0.80 (0.59‒1.08)	0.75 (0.46‒1.21)	0.83 (0.56‒1.22)	0.80 (0.57‒1.11)	1.17 (0.51‒2.71)
* *11‒14 d	0.62 (0.41‒0.96)	0.59 (0.29‒1.17)	0.61 (0.36‒1.06)	0.73 (0.44‒1.21)	0.73 (0.28‒1.91)
* *≥15 d	0.38 (0.29–0.50)	0.29 (0.19–0.45)	0.43 (0.30–0.60)	0.58 (0.42–0.80)	0.44 (0.21–0.95)
Hospital stays excluding community hospital days				Community hospital or community-based living facilities	Home
	*n* = 6339	*n* = 1549	*n* = 4790	*n* = 5969	*n* = 369
Length of stay					
* *1‒5 d	1.00 (reference)	1.00 (reference)	1.00 (reference)	1.00 (reference)	1.00 (reference)
* *6‒10 d	0.79 (0.64‒0.98)	0.82 (0.59‒1.14)	0.76 (0.57‒1.01)	0.80 (0.64‒1.00)	0.66 (0.21‒2.12)
* *11‒14 d	1.47 (1.05‒2.05)	1.50 (0.93‒2.41)	1.27 (0.79‒2.06)	1.44 (1.02‒2.04)	4.39 (0.80‒24.14)
* *≥15 d	1.07 (0.67–1.71)	0.72 (0.33–1.58)	1.45 (0.81–2.58)	1.14 (0.71–1.84)	1.19 (0.09–16.43)

*Notes*: aHR = adjusted hazard ratio; CI = confidence interval.

^a^Adjusted for age, gender, occupational socioeconomic position, university hospital catchment area, time since diagnosis of Alzheimer’s disease, type of hip fracture, admission year, place of stay at admission, use of benzodiazepines and related drugs, antipsychotics, antidepressants, history of coronary artery disease, stroke, diabetes, asthma/chronic obstructive pulmonary disease, renal failure, any cancer, and required level of assistance at the time of discharge.

## Discussion

During the follow-up, the average length of hospital stay due to hip fracture shortened an average 33 days from 2005 to 2015. Shorter length of stay in any hospital after hip fracture is associated with an increased risk of death after hospital discharge among persons with AD. Results were similar for stays including and excluding community hospital stays. Association was not linear and therefore, we examined associations by dividing length of hospital stay into fractiles.

Due to increasing health care costs and limited hospital care resources, there is a pressure for shortening hospital stays, especially in specialized settings after a major surgery. In Finnish care practice, if hip fracture patients need care and rehabilitation in hospital surroundings, they are discharged soon to community hospitals (10). Important way to shorten the hospital stay is to operate as soon as possible. Operation within 24 hours from admission to hospital was associated with lower mortality and in unadjusted analysis also with lower in-hospital pneumonia (28). In addition, staff in community hospital is used to manage persons with cognitive disorders, and to take care of common acute negative outcomes from hip fracture and procedures like delirium. In a recent study, even 42% of patients experienced postoperative delirium after hip fracture operation and in persons with cognitive disorder the odds of delirium was 3 times higher (odds ratio [OR] 2.98, 95% CI 1.74–5.14) compared to persons without cognitive disorder (29). Delirium and other postoperative complications, like surgical wound infections, other infections, and cardiovascular complications, lengthen the hospital stay (29,30), and, further, increase the risk of death (30) together with several already existing comorbidities that are exacerbated. However, we were unable to adjust our results for these factors lengthening the hospital stay because they are underreported in our data. Thus, our results may underestimate the 30-day mortality risk associated with shorter hospital stays.

Our results are in line with a recent Swedish register-based study that observed a length of stay of 1–5 days in hospital after hip fracture to double the odds of death (OR 1.97, 95% CI 1.83–2.13) after hospital discharge compared with patients with a length of stay of ≥15 days in the general population aged 50 years and older (15). Thus, the study observed a weaker association than our 4-class sensitivity analysis for the primary analysis including community hospital days (HR 2.64, 95% CI 2.01‒3.46 for 1–5 days mortality risk compared with ≥15 days, Table 3). The Swedish study observed also that the association increased through a 7-year follow-up from 2006–2012 while the average length of hospital stays decreased also in Sweden (15). Unfortunately, we did not have enough data for calendar year-specific examination of association. On the contrary, a study from the United States observed that hospital stays of more than 10 days after hip fracture increased the risk of 30-day mortality after discharge (for 11- to 14-day stays: OR 1.32, 95% CI 1.19–1.47; for ≥15-day stays: OR 2.03, 95% CI 1.84–2.24) when compared with stays of 1–5 days among hip fracture patients aged 50 years and older (16). The result is in line with our 4-class sensitivity analysis for the secondary analysis excluding community hospital days where 11- to 14-day stays had a 45% increased mortality risk (HR 1.45, 95% CI 1.04‒2.02; Table 3) when compared with stays of 1–5 days. However, we did not observe an increased death risk for ≥15-day stays compared to stays of 1–5 days. Differences in results between studies from the Nordic countries and those from the United States may arise due to differences in health care system and calculation of hospital days. In addition, treatment practices after discharge from operational hospital may be different.

To our knowledge, this is the first study that assessed the association of length of hospital stay and mortality following hip fracture in persons with AD. As persons with dementia or cognitive impairment who experience a hip fracture are more expensive to treat (31), more likely to be institutionalized (32,33), and less likely to recover function (34–36) compared with patients without cognitive impairment, their discharge, including its timing and rehabilitation, should be carefully planned. According to our study, AD patients had longer hospital stays on average after hip fracture than observed in the PERFECT study (2) among general population aged 50 years and older between 2005 and 2009. However, thereafter until 2015, the average length of hospital stay was shorter among AD patients than among the general population. It must be noted that in the PERFECT study, treatment episodes of >120 days were truncated (12) and patients admitted from long-term care were excluded, which may explain some of the differences in averages. In addition, the results from the PERFECT study are comparable to our sensitivity analyses that accounted hospital days from treatment episodes including hip fracture surgery and community hospital days. There were no differences in mortality by sex in our study. That is opposite to a previous Swedish study concerning mortality after hip fracture, in that mortality was higher among men than among women (37). The difference might be caused by differences in the populations; our study focused on AD population, and Swedish study concerned all hip fracture patients.

The strength of our study is the inclusion of all community-dwelling patients with clinically verified AD diagnosis within one country. The diagnostic protocol of AD was standardized and included rigorous exclusion diagnostics. Further, validity of the Finnish Care Register for Health Care for hip fracture is high with 98% positive predictive value and 98% coverage (38). By types of fractures, the positive predictive values are 88.1% for fractures of neck of femur, 96.0% for pertrochanteric fractures, and 62.5% for subtrochanteric fractures (38). Compared to previous studies, we provided novel information on the association of length of hospital stay after hip fracture and mortality by the place where discharged (home or community-based living facilities). We were also able to adjust for functional status at discharge.

However, our study had also some limitations. We did not have information about the time from hospital admission to operation. Those with longer waiting time to operation might have been acutely so ill that the procedure was not possible. In addition, in 2005, all hip fracture cases were newly diagnosed AD cases, whereas in later years, the population included also prevalent AD cases with longer disease durations, which hampers the comparison of different years. Missing data occurred on long hospitalizations that were ongoing at the end of follow-up on December 31, 2015; analyses did not include patients who were still in hospital at the date and may cause that length of hospital days at the most recent years seem shorter due to absence of these extreme values. We did not have data on medication use during the hospital stay. Finally, we did not have data on ASA score describing patient’s condition at the time of admission, but were able to adjust for required level of assistance at discharge that was considered as a proxy of patient’s functional status in addition to type of living at admission.

## Conclusions

Among persons with AD, shorter length of hospital stay after hip fracture was associated with an increased risk of death after discharge. After acute hip fracture treatment, inpatient rehabilitation or proper care and services in home need to be organized to older persons with AD.

## Supplementary Material

glaa199_suppl_Supplementary_Material
