# Supplementary material for: Length of Hospital Stay for Hip Fracture and 30-Day Mortality in People With Alzheimer’s Disease: A Cohort Study in Finland
Source: J Gerontol A Biol Sci Med Sci. 2020 Aug 14;75(11):2184–92. doi: 10.1093/gerona/glaa199 (PMC7566552; doi:10.1093/gerona/glaa199)
Supplement: glaa199_suppl_Supplementary_Material [file glaa199_suppl_supplementary_material.pdf]

Supplemental Material:

Length of hospital stay for hip fracture and 30-day mortality in people with Alzheimer's disease: a cohort study in Finland

Table of contents:

eFigure 1. Flow of persons with first hip fracture after diagnosis of Alzheimer's disease. Secondary analysis excluding stays in community hospitals.

eFigure 2. Association between quintiles of length of hospital stay (excluding community hospital days) after hip fracture and mortality among persons with Alzheimer's disease. Third quintile (5–6 days) as a reference group. Vertical error bars show 95% CIs.

eFigure 3. Adjusted hazard ratios for the association between 1–10 days hospital stay after hip fracture and 30-day mortality after discharge compared with >10 days hospital stay by admission years among persons with Alzheimer's disease. Vertical error bars show 95% CIs. Adjusted for age, gender, and time since diagnosis of Alzheimer's disease.

eTable 1. Definitions and classifications of covariates based on previously documented associations with fracture or death. Start of follow-up = day after the discharge date.

eTable 2. Baseline characteristics of persons with Alzheimer's disease by deciles of length of hospital stay. Proportions unless otherwise stated.

eTable 3. Association between group-specific quantiles (deciles/quintiles) of length of hospital stay after hip fracture and risk of death among persons with Alzheimer's disease.

eTable 4. Baseline (at the time of discharge from hospital care) characteristics of persons with Alzheimer's disease with first hip fracture excluding community hospital days (secondary analysis) as frequencies and proportions unless otherwise stated.

eTable 5. Number of hospital admissions, average lengths of hospital stays and proportion of deaths by admission year for persons with Alzheimer's disease with first hip fracture excluding community hospital days (secondary analysis).

eTable 6. Association between group-specific deciles of length of hospital stay after hip fracture and risk of death among persons with Alzheimer's disease in a sensitivity analysis including hospital stays that include hip fracture surgery.

eTable 7. Association between group-specific deciles of length of hospital stay after hip fracture and risk of death within 90 days after discharge among persons with Alzheimer's disease.

eTable 8. Adjusted hazard ratios for deaths occurring 27–60 days after hospital admission due to hip fracture among patients surviving at least 27 days after admission and with length of hospital stay less than 27 days.

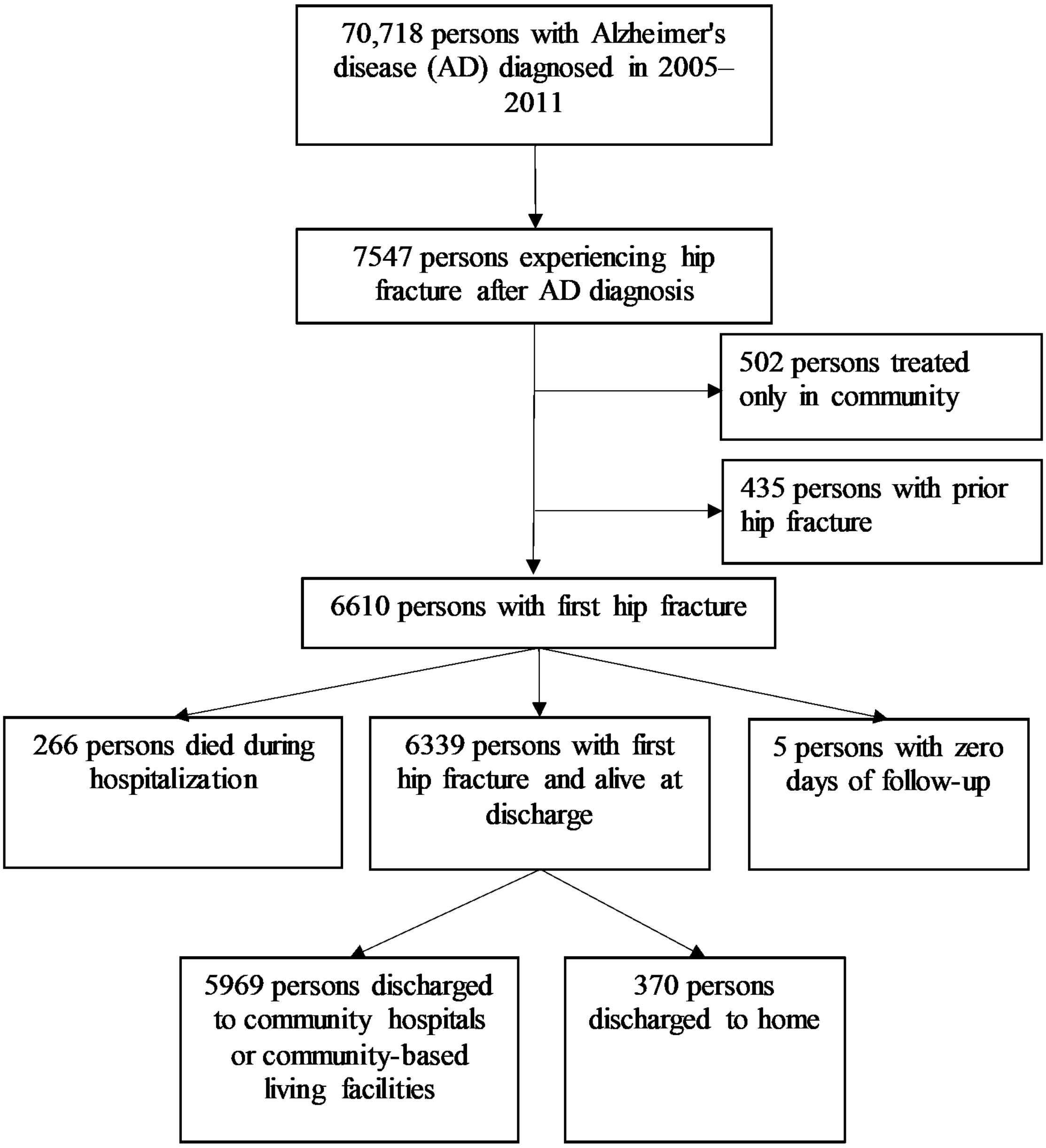

eFigure 1. Flow of persons with first hip fracture after diagnosis of Alzheimer's disease. Secondary analysis excluding stays in community hospitals.

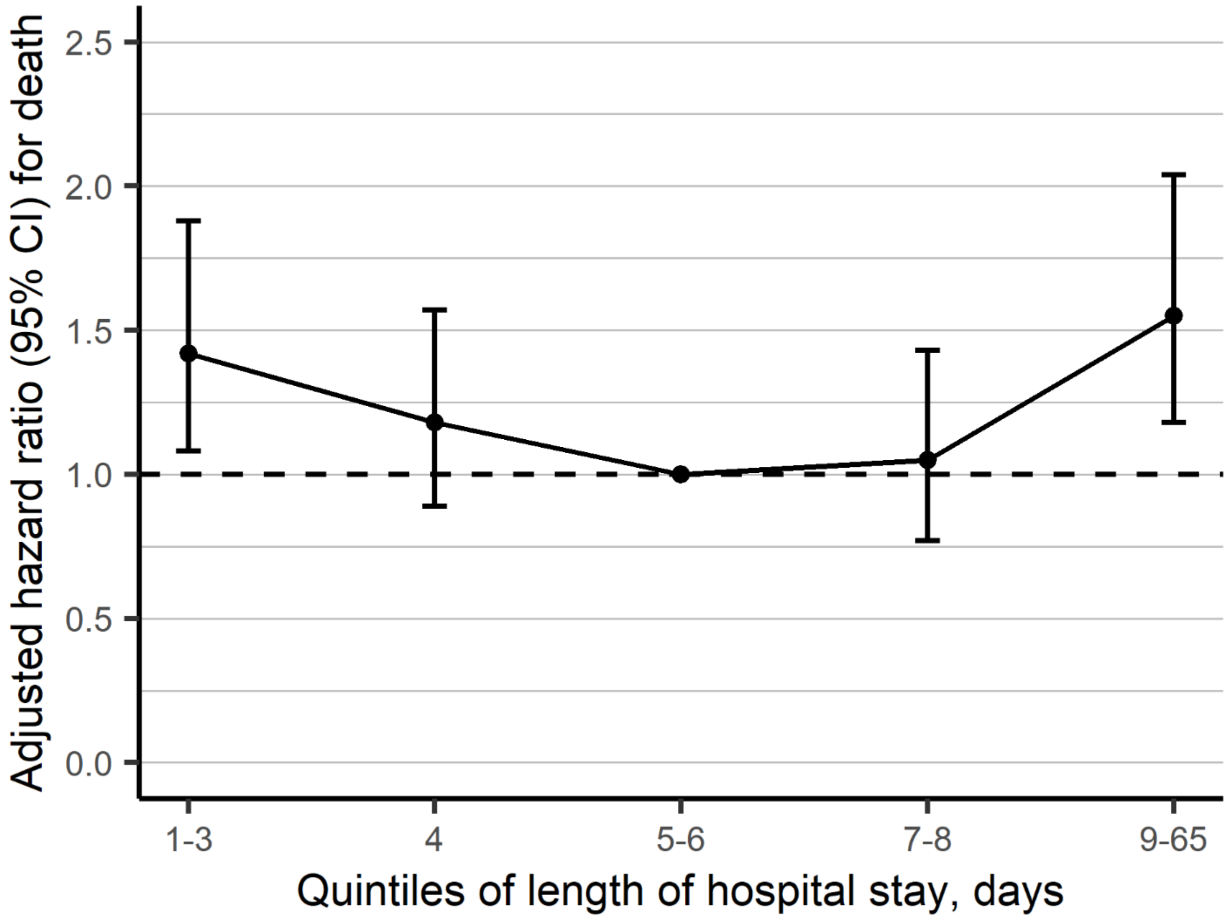

eFigure 2. Adjusted hazard ratios for the association between quintiles of length of hospital stay when excluding community hospital days after hip fracture and mortality among persons with Alzheimer's disease. Third quintile (5–6 days) as a reference group. Vertical error bars show 95% CIs. Adjusted for age, gender, occupational socioeconomic position, university hospital catchment area, time since diagnosis of Alzheimer's disease, type of hip fracture, admission year, place of stay at admission, use of benzodiazepines and related drugs, antipsychotics, antidepressants, history of coronary artery disease, stroke, diabetes, asthma/COPD, renal failure, any cancer, and required level of assistance after hospital discharge.

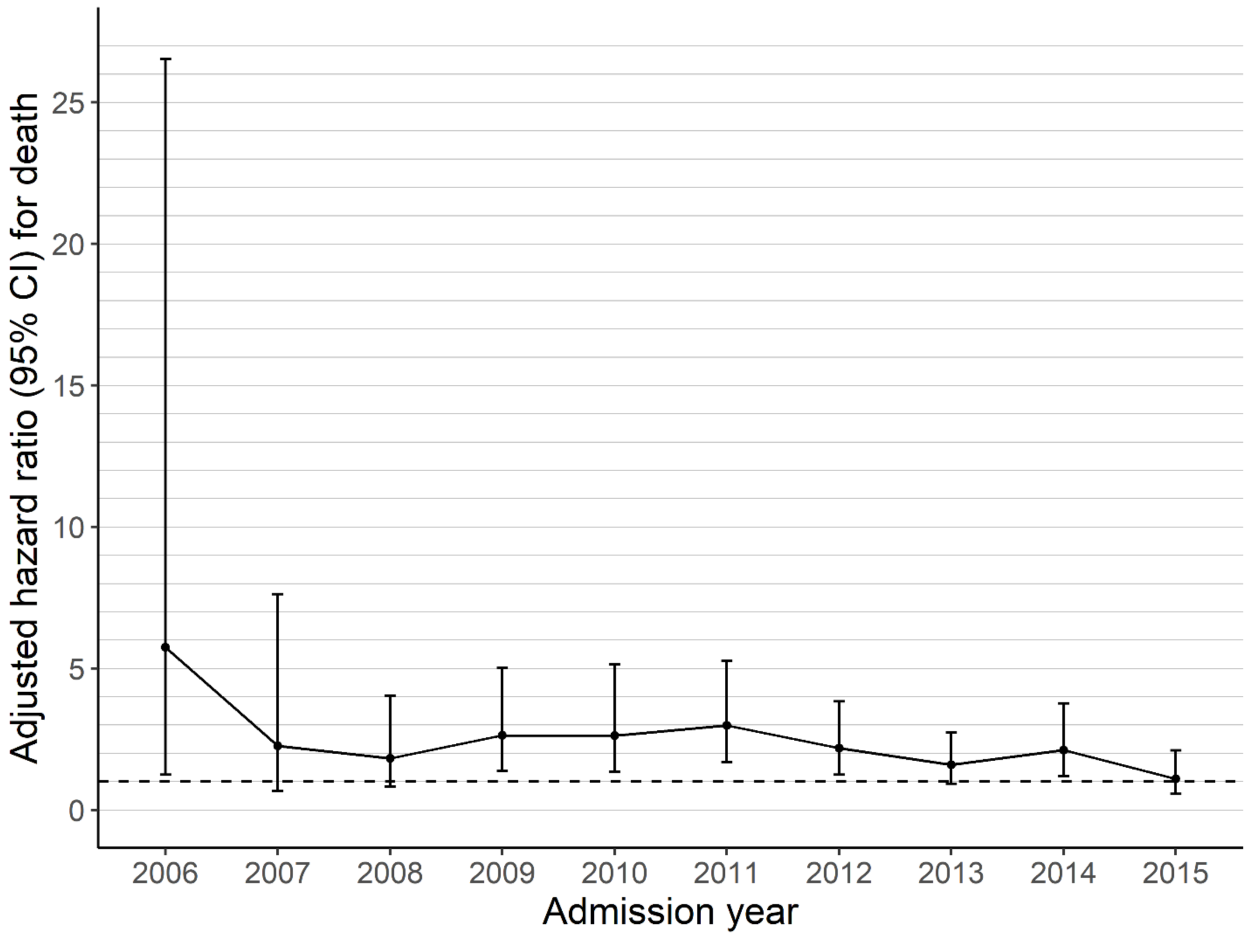

eFigure 3. Adjusted hazard ratios for the association between 1–10 days hospital stay after hip fracture and 30-day mortality after discharge compared with >10 days hospital stay by admission years among persons with Alzheimer's disease. Vertical error bars show 95% CIs. Adjusted for age, gender, and time since diagnosis of Alzheimer's disease.

eTable 1. Definitions and classifications of covariates based on previously documented associations with fracture or death. Baseline = discharge date. Start of follow-up = day after the discharge date.

**Socio-demographic and socio-economic factors**	**Classification**	**Measurement point**	**Data source**
Age, years	<75<br>75–84<br>85–	Baseline	PR
Gender	Male<br>Female	Baseline	PR
University hospital catchment area	Helsinki<br>Turku<br>Tampere<br>Kuopio<br>Oulu	Baseline	SF
Occupational socioeconomic position	Managerial/professional<br>Office<br>Farming/forestry<br>Sales, industrial, cleaning<br>Unknown and those with missing data at Statistics Finland	Highest position recorded since 1972 to 3 years prior to the diagnosis of Alzheimer's disease	SF
Time since AD diagnosis	Quartiles	From AD diagnosis date to discharge date	SRR, FCR
Admission year	2005–2015	Time of admission to hospital	FCR
Place of stay at admission	Community-based living facilities<br>Home	Time of admission to hospital	FCR
Place of stay at discharge	Community-based living facilities<br>Home	Baseline	FCR
Required level of assistance at discharge	Independent or nearly independent<br>Intermittent need<br>Recurrent need<br>Nearly continuous need<br>Continuous need<br>Unknown	Baseline	FCR
**Medication use at baseline**	**ATC code**	**Measurement period**	**Data source**
Antidepressants	N06A	Within one year prior to the start of follow-up	PR
Antipsychotics	N05A excluding lithium N05AN01	Within one year prior to the start of follow-up	PR
Benzodiazepines and related drugs	Benzodiazepines N05BA, N05CD and/or Z-drugs N05CF	Within one year prior to the start of follow-up	PR
**Cardiovascular comorbidities**	**ICD code**	**Measurement period**	**Data source**
Coronary artery disease	Hospitalization (ICD-10: I20–I25; NOMESCO: FNA, FNC, FNE, FNG00, FNG10, FN1AT, FN1BT, FN1YT) or higher medication reimbursement (206, 213, 280)	Diagnosed since 1996 until the start of follow-up in the FCR or since 1972 in the SRR	FCR, SRR
Stroke	Hospitalization (ICD-10: I60–I64, I69 ICD-9: 4330A, 4331A, 4339A, 4349A, 4340A, 4341A, 430–432, 4360, ICD-8: 430–434)	Diagnosed since 1972 until the start of follow-up	FCR

Table continues

eTable 1. Continued

Other comorbidities	ATC-code, ICD-10 code or Classification number	Measurement period	Data source
Diabetes	Diabetes medication (ATC: A10 excluding A10BX01), or higher medication reimbursement for diabetes (classification number 103)	Medication use within one year prior to the start of follow-up in the PR or diagnosed since 1972 until the start of follow-up in the SRR	PR, SRR
Asthma/COPD	Hospitalization (ICD-10: J44–J46) or higher medication reimbursement (classification number 203)	Diagnosed since 1996 until the start of follow-up in the FCR or since 1972 until the start of follow-up in the SRR	FCR, SRR
Renal failure	Hospitalization (ICD-10: I13.1, N18, N19, Z94.0, Z99.2, Z49; ICD-9: 40311, 40391, 40412, 40492, 585, 586, V420, V451, V560, V568) or higher medication reimbursement (classification numbers 137 and 138)	Diagnosed since 1996 until the start of follow-up in the FCR or diagnosed since 1972 until the start of follow-up in the SRR	FCR, SRR
Any cancer	Hospitalization (ICD-10: C00–C97, Z85; ICD-9: 140–171, 174–195, 2730, 2733, V1046, 200–208)	Diagnosed since 1987 until the start of follow-up in the FCR	FCR

Abbreviations: AD, Alzheimer's disease; ATC, Anatomical Therapeutic Chemical; COPD, chronic obstructive pulmonary disease; FCR, Finnish Care Register for Health Care; ICD, International Classification of Diseases; PR, Prescription Register; SF, Statistics Finland; SRR, Special Reimbursement Register.

eTable 2. Baseline characteristics of persons with Alzheimer's disease by deciles of length of hospital stay. Proportions unless otherwise stated.

	**1st decile (1–4 days)**	**2nd decile (5–6 days)**	**3rd decile (7–10 days)**	**4th decile (11–15 days)**	**5th decile (16–20 days)**	**6th decile (21–26 days)**	**7th decile (27–35 days)**	**8th decile (36–49 days)**	**9th decile (50–80 days)**	**10th decile (81–1544 days)**
	**n=609**	**n=583**	**n=756**	**n=556**	**n=630**	**n=593**	**n=680**	**n=626**	**n=607**	**n=630**
Age, mean (SD; years)	84.3 (6.1)	84.2 (6.8)	84.5 (6.7)	84.1 (6.3)	84.2 (6.1)	84.6 (6.1)	84.6 (5.8)	84.1 (6.3)	84.2 (5.9)	84.4 (5.5)
Age, classified, years										
<75 years	7.9	9.1	8.2	9.0	7.5	5.7	5.2	7.8	6.8	6.2
75–84 years	42.4	40.5	42.1	43.0	47.3	41.5	45.2	43.0	43.8	45.6
≥85 years	49.8	50.4	49.7	48.0	45.2	52.8	49.7	49.2	49.4	48.3
Female	74.1	75.3	77.1	74.6	79.1	79.9	77.1	76.5	79.1	75.7
Occupational socioeconomic position										
Managerial/professional	42.9	39.3	39.8	46.2	36.8	42.2	44.3	44.4	44.3	42.1
Office	20.5	16.1	14.8	17.6	23.3	17.4	17.1	16.3	15.5	17.5
Farming/forestry	7.6	11.7	12.6	8.5	10.6	9.3	11.2	9.6	7.9	9.7
Sales, industrial, cleaning	18.9	21.1	22.4	16.7	17.6	19.6	16.0	16.9	18.6	19.1
Unknown, no response	10.2	11.8	10.5	11.0	11.6	11.6	11.5	12.8	13.7	11.8
Time since diagnosis of Alzheimer's disease, mean (SD; days)	1365.5 (847.0)	1264.3 (798.1)	1214.2 (777.3)	1259.8 (809.0)	1254.1 (842.2)	1166.1 (787.2)	1127.6 (765.4)	1011.3 (746.9)	970.0 (686.5)	1085.5 (696.4)
Benzodiazepines and related drugs	30.7	31.9	31.0	37.2	38.4	36.4	35.3	34.4	32.6	27.5
Antipsychotics	37.8	35.7	35.5	36.5	35.7	35.8	34.1	32.3	26.5	28.7
Antidepressants	37.0	35.2	38.0	32.9	34.3	39.5	36.3	34.0	35.3	36.4
Coronary artery disease	32.4	30.5	27.5	26.1	30.8	29.9	30.3	32.3	29.5	34.1
Stroke	13.5	13.7	12.3	14.0	12.2	11.8	14.3	12.0	12.4	15.2
Diabetes	15.1	13.4	12.4	12.8	10.3	14.0	15.4	12.6	13.5	13.5
Asthma/COPD	11.5	10.0	8.9	11.2	11.0	11.0	12.2	10.5	10.4	11.6
Renal failure	1.5	1.5	1.6	1.3	2.4	0.8	1.5	1.6	1.7	1.6
Any cancer	14.6	13.7	12.7	11.5	12.7	13.2	15.6	15.3	13.7	16.7

Table continues

eTable 2. Continued

	1st decile (1–4 days)	2nd decile (5–6 days)	3rd decile (7–10 days)	4th decile (11–15 days)	5th decile (16–20 days)	6th decile (21–26 days)	7th decile (27–35 days)	8th decile (36–49 days)	9th decile (50–80 days)	10th decile (81–1544 days)
	**n=609**	**n=583**	**n=756**	**n=556**	**n=630**	**n=593**	**n=680**	**n=626**	**n=607**	**n=630**
Required level of assistance										
Independent or nearly independent	1.8	5.8	5.8	4.5	5.4	8.1	4.7	5.4	3.5	2.1
Intermittent need	10.3	14.2	11.5	13.3	20.8	21.6	25.6	20.5	22.2	10.3
Recurrent need	38.1	36.2	42.6	32.7	31.6	33.9	29.0	37.9	37.9	33.3
Nearly continuous need	22.7	21.6	17.6	17.3	17.8	13.5	15.0	14.5	14.8	21.1
Continuous need	14.6	13.7	19.4	28.1	21.3	19.2	22.9	19.8	20.1	31.1
Unknown	12.5	8.4	3.0	4.1	3.2	3.7	2.8	1.9	1.5	2.1
Type of hip fracture										
Fracture of neck of femur	61.6	58.7	65.6	70.5	67.1	68.8	61.5	58.3	55.0	56.2
Pertrochanteric fracture	32.8	35.3	28.6	25.0	28.4	25.1	33.4	35.0	36.6	35.7
Subtrochanteric fracture	5.6	6.0	5.8	4.5	4.4	6.1	5.1	6.7	8.4	8.1
Place of stay at admission										
Community-based living facilities	25.0	21.6	26.2	27.3	21.6	24.1	21.9	21.4	21.6	23.5
Home	75.0	78.4	73.8	72.7	78.4	75.9	78.1	78.6	78.4	76.5
Place of stay at discharge										
Community-based living facilities	84.1	78.7	68.9	48.6	35.1	28.3	24.1	24.0	28.5	49.7
Home	15.9	21.3	30.8	51.4	64.6	71.5	75.3	76.0	71.2	50.3
Missing data			0.3		0.3	0.2	0.6		0.3	

eTable 3. Association between group-specific quantiles (deciles/quintiles) of length of hospital stay after hip fracture and risk of death among persons with Alzheimer's disease.

Primary analysis	Persons with first hip fracture **including** community hospital days (n=6270)		Secondary analysis	Persons with first hip fracture **excluding** community hospital days (n=6339)	
	Unadjusted HR (95% CI)	Adjusted[a] HR (95% CI)		Unadjusted HR (95% CI)	Adjusted[a] HR (95% CI)
Deciles of length of hospital stay			Quintiles of length of hospital stay		
1st (1–4 days)	3.11 (1.87–5.16)	2.76 (1.66– 4.60)	1st (1–3 days)	1.65 (1.26–2.17)	1.42 (1.08–1.88)
2nd (5–6 days)	2.74 (1.63–4.58)	2.52 (1.50–4.23)	2nd (4 days)	1.28 (0.97–1.69)	1.18 (0.89–1.57)
3rd (7–10 days)	2.50 (1.51–4.14)	2.22 (1.34–3.69)	3rd (5–6 days)	1.00 (reference)	1.00 (reference)
4th (11–15 days)	1.85 (1.06–3.21)	1.58 (0.91–2.75)	4th (7–8 days)	1.00 (0.74–1.36)	1.05 (0.77–1.43)
5th (16–20 days)	1.63 (0.94–2.83)	1.55 (0.89–2.70)	5th (9–65 days)	1.54 (1.18–2.01)	1.55 (1.18–2.04)
6th (21–26 days)	1.00 (reference)	1.00 (reference)			
7th (27–35 days)	1.05 (0.58–1.90)	0.99 (0.55–1.79)			
8th (36–49 days)	1.14 (0.63–2.07)	1.10 (0.60–1.99)			
9th (50–80 days)	1.08 (0.59–1.98)	1.05 (0.57–1.93)			
10th (81–1544 days)	0.99 (0.54–1.82)	0.73 (0.39–1.36)			

[a]Adjusted for age, gender, occupational socioeconomic position, university hospital catchment area, time since diagnosis of Alzheimer's disease, type of hip fracture, admission year, place of stay at admission, use of benzodiazepines and related drugs, antipsychotics, antidepressants, history of coronary artery disease, stroke, diabetes, asthma/COPD, renal failure, any cancer, and required level of assistance after hospital discharge.

eTable 4. Baseline (at the time of discharge from hospital care) characteristics of persons with Alzheimer's disease with first hip fracture excluding community hospital days (secondary analysis) as frequencies and proportions unless otherwise stated.

	**Persons with first hip fracture (n=6 605)**	**Persons died during hospital stay (n=266)**	**Persons alive at discharge (n=6 339)**	**Discharged to community hospital or community-based living facilities (n=5969)**	**Discharged to home (n=370)**
	n (%)	n (%)	n (%)	n (%)	n (%)
Age, mean (SD; years)	84.5 (6.2)	86.8 (5.1)	84.4 (6.2)	84.4 (6.1)	83.8 (7.0)
Age, classified, years					
<75 years	464 (7.0)	4 (1.5)	460 (7.3)	420 (7.0)	40 (10.8)
75–84 years	2855 (43.2)	85 (32.0)	2770 (43.7)	2616 (43.8)	154 (41.7)
≥85 years	3286 (49.8)	177 (66.5)	3109 (49.0)	2933 (49.1)	175 (47.4)
Female	4939 (74.8)	149 (56.0)	4790 (75.6)	4489 (75.2)	300 (81.3)
Occupational socioeconomic position					
Managerial/professional	2797 (42.3)	115 (43.2)	2682 (42.3)	2537 (42.5)	145 (39.3)
Office	1188 (18.0)	56 (21.1)	1132 (17.9)	1075 (18.0)	56 (15.2)
Farming/forestry	633 (9.6)	19 (7.1)	614 (9.7)	576 (9.7)	38 (10.3)
Sales, industrial, cleaning	1233 (18.7)	45 (16.9)	1188 (18.7)	1092 (18.3)	96 (26.0)
Unknown, no response	754 (11.4)	31 (11.7)	723 (11.4)	689 (11.5)	34 (9.2)
Time since diagnosis of Alzheimer's disease, mean (SD; days)	1153.4 (783.6)	1151.9 (783.8)	1153.4 (783.6)	1136.0 (778.6)	1433.7 (812.9)
Benzodiazepines and related drugs	2211 (33.5)	89 (33.5)	2122 (33.5)	1995 (33.4)	127 (34.4)
Antipsychotics	2243 (34.0)	85 (32.0)	2158 (34.0)	2015 (33.8)	143 (38.8)
Antidepressants	2343 (35.5)	87 (32.7)	2256 (35.6)	2102 (35.2)	154 (41.7)
Coronary artery disease	2047 (31.0)	118 (44.6)	1929 (30.4)	1841 (30.8)	88 (23.9)
Stroke	890 (13.5)	48 (18.1)	842 (13.3)	791 (13.3)	51 (13.8)
Diabetes	883 (13.4)	38 (14.3)	845 (13.3)	808 (13.5)	37 (10.0)
Asthma/COPD	739 (11.2)	47 (17.7)	692 (10.9)	662 (11.1)	30 (8.1)
Renal failure	103 (1.6)	6 (2.3)	97 (1.5)	91 (1.5)	6 (1.6)
Any cancer	938 (14.2)	41 (15.4)	897 (14.2)	843 (14.1)	53 (14.4)

Table continues

eTable 4. Continued

	Persons with first hip fracture (n=6 605)	Persons died during hospital stay (n=266)	Persons alive at discharge (n=6 339)	Discharged to community hospital or community-based living facilities (n=5969)	Discharged to home (n=370)
	n (%)	n (%)	n (%)	n (%)	n (%)
Required level of assistance					
Independent or nearly independent	133 (2.0)	0	133 (2.1)	70 (1.2)	63 (17.1)
Intermittent need	779 (11.8)	0	779 (12.3)	697 (11.7)	82 (22.2)
Recurrent need	2896 (43.9)	1 (0.4)	2895 (45.7)	2788 (46.7)	107 (29.0)
Nearly continuous need	1410 (21.4)	4 (1.5)	1406 (22.2)	1354 (22.7)	52 (14.1)
Continuous need	707 (10.7)	0	707 (11.1)	671 (11.2)	36 (9.8)
Unknown	680 (10.3)	261 (98.1)	419 (6.6)	389 (6.5)	30 (7.9)
Type of hip fracture					
Fracture of neck of femur	4069 (61.6)	172 (64.7)	3919 (61.4)	3648 (61.1)	248 (67.2)
Pertrochanteric fracture	2134 (32.3)	81 (30.4)	2068 (32.4)	1950 (32.7)	103 (27.9)
Subtrochanteric fracture	402 (6.1)	13 (4.9)	393 (6.2)	371 (6.2)	18 (4.9)
Place of stay at admission					
Community-based living facilities	1560 (23.6)	65 (24.4)	1495 (23.6)	1426 (23.9)	68 (18.4)
Home	5045 (76.4)	201 (75.6)	4844 (76.4)	4543 (76.1)	301 (81.6)

eTable 5. Number of hospital admissions, average lengths of hospital stays and proportion of deaths by admission year for persons with Alzheimer's disease with first hip fracture excluding community hospital days (secondary analysis).

Admission year	2005	2006	2007	2008	2009	2010	2011	2012	2013	2014	2015	Total
**Among persons who died during hospital stay (n=266)**												
N of admissions	0	14	18	22	38	33	28	31	29	25	28	266
Mean (SD) LOS (days)	–	4.6 (3.2)	5.2 (2.6)	6.2 (5.5)	5.7 (4.5)	6.5 (4.4)	4.1 (2.1)	5.4 (4.9)	5.1 (4.2)	6.4 (7.1)	5.2 (4.3)	5.5 (4.5)
Median (IQR) LOS (days)	–	3 (3–5)	4.5 (3–7)	5 (3–7)	5 (3–6)	5 (3–10)	4 (2–5)	3 (2–6)	3 (2–6)	4 (2–8)	3.5 (2.5–5.5)	4 (3–6)
**Among persons alive at discharge (n=6339)**												
N of admissions	64	236	351	548	602	759	923	908	813	622	513	6339
Mean (SD) LOS (days)	8.8 (5.6)	8.7 (7.2)	7.5 (4.1)	7.2 (4.6)	6.9 (3.8)	7.2 (5.0)	6.6 (4.0)	6.0 (3.1)	5.6 (3.1)	5.4 (3.3)	5.2 (2.9)	6.4 (4.1)
Median (IQR) LOS (days)	7 (5.5–10)	6.5 (5–10)	6 (5–9)	6 (4–9)	6 (4–8)	6 (4–8)	6 (4–8)	5 (4–7)	5 (4–7)	5 (4–6)	4 (3–6)	5 (4–8)
N (%) of deaths within 30 days after discharge	1 (1.6)	9 (3.8)	17 (4.8)	29 (5.3)	47 (7.8)	56 (7.4)	78 (8.5)	65 (7.2)	69 (8.5)	63 (10.1)	42 (8.2)	476 (7.5)
**Among persons alive at discharge who were discharged to community hospital or community-based living facilities (n=5969)**												
N of admissions	61	231	337	533	582	717	875	859	750	563	461	5969
Mean (SD) LOS (days)	8.7 (5.7)	8.7 (7.3)	7.3 (4.0)	7.0 (4.0)	6.8 (3.7)	6.9 (4.2)	6.4 (3.3)	5.9 (3.0)	5.5 (3.1)	5.4 (3.4)	5.2 (3.0)	6.3 (3.8)
Median (IQR) LOS (days)	7 (5–10)	6 (5–10)	6 (5–9)	6 (4–8)	6 (4–8)	6 (4–8)	6 (4–8)	5 (4–7)	5 (4–6)	4 (3–6)	4 (3–6)	5 (4–8)
N (%) of deaths within 30 days after discharge	1 (1.6)	9 (3.9)	17 (5.0)	29 (5.4)	45 (7.7)	55 (7.7)	74 (8.5)	64 (7.5)	66 (8.8)	59 (10.5)	36 (7.8)	455 (7.6)

Table continues

eTable 5. Continued

**Admission year**	**2005**	**2006**	**2007**	**2008**	**2009**	**2010**	**2011**	**2012**	**2013**	**2014**	**2015**	**Total**
**Among persons alive at discharge who were discharged to home (n=369)**												
N of admissions	3	5	14	15	20	43	48	49	63	59	51	369
Mean (SD) LOS (days)	57.7 (55.0)	47.0 (32.9)	48.0 (37.4)	45.0 (37.9)	46.1 (38.7)	43.2 (42.2)	40.0 (52.6)	42.5 (49.3)	36.3 (43.7)	33.1 (46.8)	22.8 (21.1)	39.7 (43.9)
Median (IQR) LOS (days)	8 (7–12)	7 (7–10)	8 (6–15)	10 (7–13)	7 (5.5–10.5)	8 (5–11)	6 (5–10)	7 (5–8)	6 (4–7)	5 (4–7)	5 (3–6)	6 (5–8)
N (%) of deaths within 30 days after discharge	0 (0.0)	0 (0.0)	0	0	2 (10.0)	1 (2.4)	4 (8.3)	1 (2.0)	3 (4.8)	4 (6.8)	6 (11.8)	21 (5.7)

Note. Abbreviations: LOS, length of stay.

eTable 6. Association between group-specific deciles of length of hospital stay after hip fracture and risk of death among persons with Alzheimer's disease in a sensitivity analysis including hospital stays that include hip fracture surgery.

	Persons with first hip fracture: **Hospital stays including a hip fracture surgery and days in community hospitals** (n=5604)	
Deciles of length of hospital stay	Unadjusted HR (95% CI)	Adjusted[a] HR (95% CI)
1st (1–4 days)	3.21 (1.76–5.83)	2.80 (1.53–5.12)
2nd (5–7 days)	3.37 (1.92–5.90)	3.09 (1.76–5.44)
3rd (8–10 days)	2.93 (1.61–5.33)	2.45 (1.34–4.49)
4th (11–16 days)	2.10 (1.15–3.85)	1.86 (1.01–3.42)
5th (17–21 days)	1.78 (0.95–3.34)	1.71 (0.91–3.22)
6th (22–27 days)	1.00 (reference)	1.00 (reference)
7th (28–35 days)	1.47 (0.76–2.84)	1.40 (0.73–2.71)
8th (36–49 days)	1.32 (0.68–2.55)	1.28 (0.66–2.49)
9th (50–81 days)	1.38 (0.71–2.68)	1.35 (0.69–2.62)
10th (82–1544 days)	1.22 (0.62–2.41)	0.95 (0.48–1.87)

[a]Adjusted for age, gender, occupational socioeconomic position, university hospital catchment area, time since diagnosis of Alzheimer's disease, type of hip fracture, admission year, place of stay at admission, use of benzodiazepines and related drugs, antipsychotics, antidepressants, history of coronary artery disease, stroke, diabetes, asthma/COPD, renal failure, any cancer, and required level of assistance at the time of discharge.

eTable 7. Association between group-specific deciles of length of hospital stay after hip fracture and risk of death within 90 days after discharge among persons with Alzheimer's disease.

	Persons with first hip fracture (n=6270)	
Deciles of length of hospital stay	Unadjusted HR (95% CI)	Adjusted[a] HR (95% CI)
1st (1–4 days)	2.46 (1.76–3.43)	2.28 (1.63–3.19)
2nd (5–6 days)	2.03 (1.44–3.86)	1.96 (1.39–2.77)
3rd (7–10 days)	2.24 (1.62–3.09)	2.12 (1.53–2.94)
4th (11–15 days)	1.56 (1.09–2.24)	1.41 (0.98–2.03)
5th (16–20 days)	1.29 (0.90–1.86)	1.26 (0.87–1.82)
6th (21–26 days)	1.00 (reference)	1.00 (reference)
7th (27–35 days)	1.12 (0.77–1.62)	1.08 (0.74–1.56)
8th (36–49 days)	1.03 (0.70–1.51)	1.03 (0.70–1.51)
9th (50–80 days)	0.80 (0.53–1.21)	0.81 (0.54–1.23)
10th (81–1544 days)	0.94 (0.63–1.39)	0.76 (0.51–1.14)

[a]Adjusted for age, gender, occupational socioeconomic position, university hospital catchment area, time since diagnosis of Alzheimer's disease, type of hip fracture, admission year, place of stay at admission, use of benzodiazepines and related drugs, antipsychotics, antidepressants, history of coronary artery disease, stroke, diabetes, asthma/COPD, renal failure, any cancer, and required level of assistance at the time of discharge.

eTable 8. Adjusted hazard ratios for deaths occurring 27–60 days after hospital admission due to hip fracture among patients surviving at least 27 days after admission and with length of hospital stay less than 27 days.

	Patients surviving at least 27 days after admission and with length of hospital stay less than 27 days (n=3334 with 206 deaths)	
	Unadjusted HR (95% CI)	Adjusted[a] HR (95% CI)
Length of stay		
1–4 days	1.78 (1.09–2.91)	1.71 (1.02–2.86)
5–6 days	1.68 (1.02–2.75)	1.66 (1.00–2.78)
7–10 days	1.63 (1.01–2.61)	1.64 (1.01–2.68)
11–15 days	1.20 (0.71–2.05)	1.06 (0.61–1.84)
16–20 days	1.20 (0.71–2.01)	1.14 (0.67–1.93)
21–26 days	1.00 (reference)	1.00 (reference)

[a]Adjusted for age, gender, occupational socioeconomic position, university hospital catchment area, time since diagnosis of Alzheimer's disease, type of hip fracture, admission year, place of stay at admission, use of benzodiazepines and related drugs, antipsychotics, antidepressants, history of coronary artery disease, stroke, diabetes, asthma/COPD, renal failure, any cancer, and required level of assistance after hospital discharge.
